# The predictors of unsuppressed viremia among PLHIV: a cross-sectional study in Ghana

**DOI:** 10.1186/s12889-023-16032-9

**Published:** 2023-06-09

**Authors:** Lydia Boampong Owusu, Christiana Ababio, Selina Boahene, Abdul-Fatawu Suglo Zakaria, Abigael Omowumi Emikpe, Catherine Kroamah Dwumfour, Kofi Antwi Appiagyei, Felix Apiribu

**Affiliations:** 1 Kwame Nkrumah University of Science and Technology, Kumasi, Ghana; 2 New Edubiase Government Hospital, Adansi South District, Ashanti Region, Ghana; 3 Nurses and Midwives Training College, Tamale, Ghana

**Keywords:** HIV, AIDS, People living with HIV, AIDS, Viral load, Non-suppression, Antiretroviral therapy

## Abstract

**Background:**

Unsuppressed viremia in HIV infected patients is generally associated with increased rates of disease transmission and poor patient survival. This study assessed the socio-demographic determinants of People Living with HIV/AIDS, having viral load non-suppression and who are receiving antiretroviral therapy in a District Hospital in Ghana.

**Methods:**

The study utilized the cross-sectional research design with both primary and secondary data conducted from September to October 2021 in Ghana. Data were collected from 331 PLHIV who were placed on Anti-Retroviral Therapy (ART) for more than 12 months at the ART centre at a District Hospital in Ghana. Unsuppressed viremia was defined as plasma viral load of ≥ 1000 copies/mL after 12 months on an ART with effective adherent support. A structured questionnaire was used to collect primary data on participants and a Secondary data was also collected from patients’ folders, hospital registers and the computerized health information systems at the study site. SPSS was used to analyse descriptive and inferential data. Pearson’s chi-square and Fisher’s exact test were used to assess the independent determinants of viral load non-suppression. Pearson’s chi-square test was used for tests giving ≤ 20% of expected cell counts less than five while Fisher’s exact test was used for tests giving > 20% of expected cell counts less than five. A *p* value of < 0.05 was considered statistically significant.

**Results:**

Out of the 331 PLHIV who participated in the study, 174 (53%) were female and 157 (47%) were Male. The study found viral load non-suppression of 19% with age (*p* = 0.03), income (*p* = 0.02), employment (*p* = 0.04), means of transportation (*p* = 0.02), cost of transportation to the ART centre (*p* = 0.03) and level of medication adherence (*p* = 0.02) as determinants of viral load non-suppression.

**Conclusion:**

There was a low level of viral load non-suppression among PLHIV after 12 months of active antiretroviral therapy with age, income, employment, means of transportation, cost of transportation and level of medication adherence influencing viral non-suppression. Thus, ART drugs and services should be decentralized to the community health workers’ level within the various localities of patients to decrease the economic consequences involved in accessing health care for PLHIV/AIDS. This will minimize defaulting, improve adherence and promote viral load suppression.

## Introduction

Global HIV Statistics in 2019, estimated the burden of Human Immunodeficiency Virus (HIV) infection to be 37.9 (32.7 million – 44.0 million) million people who are living with the virus. There are 36.2 (31.3 million – 42.0 million) million adults and 1.7 (1.4 million – 2.3 Million) million children (< 15 years) who are currently living with the HIV pandemic globally, with 25.6 (22.2 – 29.5 million) million of the total burden living in Sub-Saharan Africa (SSA) [1]. The African region accommodates nearly two thirds (25.7 million) of the global population of the HIV infection pandemic [1]. Ghana has an HIV prevalence of 1.69% with Ahafo Region having the highest HIV prevalence of 2.66% and Eastern region the lowest prevalence of 0.39% [2].

The USAID 95-95-95 target to end AIDS states that by 2030, 95% of people living with HIV know their HIV status, 95% of people living with HIV and who know their HIV status are on antiretroviral therapy and 95% of people who are on antiretroviral therapy have suppressed viral loads [3]. Access to antiretroviral therapy (ART) by people living with HIV and Acquired Immunodeficiency Syndrome (AIDS) has gradually seen an increase over the years with 62% adult and 54% children coverage internationally. In 2020, an estimated 37.7 million people were living with HIV globally and 73% of these people had received ART [4]. This has positively changed the dynamics of HIV infection. Between 2000 – 2018, the number of new HIV infections reduced by 37% and HIV/AIDS-related mortality also reduced by 45% with over 13.6 million lives saved due to ART [5]. At the end of 2018, an estimated 79% of all people living with HIV were aware of their status with 62% receiving highly active life-saving ART and 53% achieving viral suppression [5].

The essence of ART is to suppress the replication of HIV to the barest minimal level, halt the development of drug resistance viruses, restore and improve patient’s immunity, combat HIV/AIDS-related events and enhance the life span of the HIV-infected individual [6–8]. Successful ART is determined by low or undetected viral load count (the number of RNA copies of the virus per millilitre of patient’s blood sample, that is < 1000 copies/ml) [9]. Viral load monitoring increases life expectancy [10, 11], and ensures a reduction in misdiagnosis of treatment failure, leading to effective utilization of limited resources [9]. At the individual level, the morbidity and mortality of the virus are reduced by adherence to ART [12, 13], thereby increasing the likelihood of individual patients recovering their immunity and reducing the chances of sexually transmitting the HIV to the seronegative partner. At the population level HIV incidence is reduced and offers the hope of ending the HIV/AIDS epidemic as envisioned by the United Nations Organization [11, 13].

The Ghana AIDS Commission has estimated that about 43% of patients on ART have achieved viral load suppression as of 2019 and there are a number of factors leading to viral non-suppression among patients on ART including; being an adolescent, nutritional scarcity, income and other economic factors [14, 15]. It is also of much importance to identify the factors that reduce adherence to antiretroviral therapy for people living with HIV hence causing viral non-suppression [16]. This study, therefore, assessed the proportion of PLHIV who are on ART and are viral load non-suppressed and the socio-demographic determinants of viral load non-suppression among PLHIV/AIDS on active anti-retroviral therapy in Adansi South District in Ghana. The study is necessitated by the threat and the complexity of HIV transmission from people living with HIV and AIDS (PLHIVA) to their seronegative partners, mother to child transmission, PLHIV/AIDs to their caregivers and the endless chain of infection since the only way to achieve viral load suppression is to achieve ART adherence. The findings will be used to make recommendations that will result in viral load suppression.

## Materials and methods

### Study design and setting

A descriptive cross-sectional design using the quantitative approach was utilized to assess the determinants of viral non-suppression among 331 PLHIV who are on ART for over 12 months at the ART outpatient clinic at the District Government Hospital in the Adansi South District in Ghana.

### Study population

The study was conducted in the District Government Hospital located in the Adansi South District in Ashanti Region. It has an Accident & Emergency centre, Laboratory services centre, Eye Clinic, Pharmacy, Antenatal centre, Obstetrics/ Gynaecology services, Mental health, Hypertension and Diabetes centres, and Reproductive and Child health centres. The facility has a daily OPD attendance of over 150 and an annual attendance between 10,000 and 15,000. Adansi South District was created on 18^th^ February 2004 by a Legislative Instrument (LI 1752) through an Act of parliament (Act 462, 1993), after being carved out of Adansi West District and Adansi East District Assemblies in the same year. The district capital, New Edubiase is about 92 km from Kumasi, the regional capital, along the Cape Coast – Kumasi trunk road. The total population of Adansi South District, according to 2021 Population and Housing Census is 85,200 with 42,552 males and 42,648 females [17].

The Adansi South district was selected for the study because the district has been in the news in Ghana in terms of HIV/AIDS infection since 2005 with an HIV prevalence of 3.7% as at 2005 [18]. As at 2019, the Ashanti region where the district is located had an HIV/AIDS adult prevalence of 1.94% with the national adult prevalence of 1.7% [19]. The Adansi South District in 2021 inaugurated an HIV/AIDS committee to help reduce the spread of HIV/AIDS [20]. This made the researchers more curious to know the state of HIV/AIDS infection in the district. The district hospital was used because it is the main site of ART distribution and centre for HIV care in the district.

The study population included all HIV-infected adults ≥ 18 years who had been on antiretroviral therapy and had attended the clinic at the selected study site in Adansi South from January 2018 to October 2021. The study included all PLHIVs who were 18 years and above and have been on ARTs for 12 months and above at the selected ART clinic at the study site. Data from the District Hospital showed that 3 out of 329 PLHIV who initiated ART in 2018 died, two out of 469 PLHIV receiving ART died in 2019, four out of 502 PLHIV receiving ART died in 2020 and six out of 615 PLHIV on ART died in 2021. Thus, a population of 1900 was used in the sample size calculation.

### Sample size calculation and sampling procedure

Yamane’s formula for calculating sample size was used to determine the total sample size as stated below:

$$\mathrm{n}=\frac{\mathrm{N}}{\left(1+\mathrm{N }{\left(\mathrm{e}\right)}^{2}\right)}$$

where, n = sample size

N = population under study

e = margin of error (0.05)

Thus,

$$\mathrm{n}=\frac{1900}{\left(1+1900 {\left(0.05\right)}^{2}\right)}$$

$$\mathrm{n}= 331$$

At 20% non-response rate = 66.2

Sample size = 398, however due to resource constraints, a final sample size of 331 was used for the study.

A total of 331 folders of patients who satisfied the inclusion criteria were sampled. 331 PLHIV participated in the study. For this study it was assumed that PLHIV are well aware of viral loads and its importance thus, they will be willing to discuss this subject to help draw conclusion to identify determinants of viral load non-suppression.

The variable gender in this study refers to male or female and was measured according to the participants’ choice on the questionnaire. Marital status considers the current status of the participant in terms of intimate relationship with a spouse or prospective partner, termination of this relationship or the demise of a partner. Marital status was measured by participants’ choice of responses as itemised on the questionnaire. A person is considered married when customary rites have been performed according to Ghana laws or a law court has registered their marriage. Again, ‘divorced’ means separation by law while ‘separated’ was considered as one spouse walking away from marriage. ‘Widowed’ was considered as the demise of a spouse. ‘Single’ status was considered for persons not involved in any intimate relationship while cohabiting refers to persons living together as partners in the absence of legal or customary marriage. There were three categories of religion based on the system in Ghana. Christians are those known to believe in Jesus Christ and worship in churches while Muslims are those who believe in Muhammed and worship in mosques. Traditionalists are those who worship smaller gods in shrines. These were self-reported.

Educational status was categorised into five groups. Those without formal education included participants who had never enrolled into any kind of educational centre registered with the Ghana Education Service. Primary denotes those who had attained basic education and JHS status involves persons who enrolled or have taken the Basic Education Certificate Examination. SHS status was assigned to those who enrolled or had taken the West African Senior School Certificate Examination while tertiary status involved those enrolled in an accredited university or training college. These were all self-reported. Employed status include those who have gained positions or roles in government or private companies to perform assigned duties. Those involved in personal business were also considered employed. Those without jobs whether in the government or public sector or personal business including students were considered unemployed. Age means the length of time a person has lived on earth. Monthly income, means of transport and cost of travelling to ART centre were self-reported. Transport by bus included those with private cars or motors and those who joined public buses, motors or tricycles to their destination. While those on foot included participants who walked to the ART centres.

Patients with plasma viral load of ≥ 1000 copies/mL after 12 months on an ART with effective adherent support were considered as viral non-suppressed, those with plasma viral load of < 1000 copies/mL after being on ART as viral suppressed and patients with plasma viral load of < 400 copies/mL after being on ART were considered as undetectable. Participants were grouped into good adherence and poor adherence. Good adherence referred to strict compliance or not missing pills in their history while poor adherence referred to non-compliance or missing pills sometimes or often in their history.

### Sampling

A random sampling method was employed in this study. Outpatients who met the inclusion criteria were asked to select Yes or No labelled pieces of papers randomly from a concealed box. Those who picked yes were included in the study. The process was repeated until the sample size of 331 had been achieved. Those who picked yes were taken through detailed information session on the study, their questions were answered, and they were taken through informed consent form signing.

### Data collection procedure

An approval letter was sought from the District Government Hospital in the Adansi South District in Ghana. Permission was sought from the Unit in charge at the ART centre, thereafter patients were approached, and the purpose of the study was explained to them. Patients who consented to the study were given the questionnaire to fill. A structured questionnaire was used in collecting data on independent variables such as sex, age, marital status, religion, educational status, occupation, monthly income, means of transport, cost of travelling to ART centre, duration on ART to determine the socio-demographic characteristics of participants. Data on viral loads of participants were recorded as the dependent variable of this study. All variables were self-reported with the exception of viral loads which were recorded from patients’ folders and records. Upon their consent, primary data were collected from the patients on monthly income, means and cost of transportation to ART centre as well as time to reach the ART Centre were obtained from the consented patients with a questionnaire. Afterwards, Secondary data was collected from the patients’ folders, hospital registers and the computerized health information systems at the study site. The tool collected data on the socio-demographic as well as clinical data of the patients to meet the study objectives. Three nurses who served as research assistants were trained to collect the data over a period of 28 days between September to October 2021. Viral loads of PLHIV were obtained by collecting and labelling blood samples and transporting to the largest referral hospital in the Region, Komfo Anokye Teaching Hospital for analysis.

### Data management and data quality assurance methods

Data was encrypted and stored on a secure cloud storage which is in line with Ghana’s Data Protection Act subsection 24. Soft copy data was backed up on an encrypted online storage to avoid data loss. At the end of data collection, data was transferred into Microsoft excel and encrypted for added security. The Microsoft excel file was only available on a password protected computer accessible to the research team. Hard copies of filled questionnaire were sealed in an envelope and kept under lock and key in a locker in the principal researcher’s office. Data quality assurance was done by double entry of results by two different people for validation. Both hard copy data and online data were merged after data entry. The data was cleaned to correct mistakes, assess missing data and determine appropriate methods for resolving challenges including follow-ups. All methods were carried out in accordance with relevant guidelines.

### Data analysis

Data were analysed using a statistical package for social sciences (SPSS) version 20. Descriptive statistics: frequencies were used to describe demographic and socioeconomic related characteristics of the participants. The data were presented using tables, flow charts, pie charts, diagrams, and graphs among others to communicate effectively. Pearson’s chi-square and Fisher’s exact test were used to test the association between selected independent variables and unsuppressed viremia as the dependent variable. Test statistics with a p-value of < 0.05 were considered statistically significant.

## Results

A total of three hundred and thirty-one (331) Secondary data of HIV patients who were on ART and met the inclusion criteria were retrieved from the ART Centre and used for the analysis.

### Demographic characteristics of participants

One hundred and seventy-four (53%) of the participants were females. Age distribution was even across the groups. Most of the participants (64%) were married, with 42 (13%) of them separated and two (7%) widowed. For the religious affiliation of the participants, most of them were from the Christian faith 223 (67.4%). With regards to educational attainment, 49 (15%) did not have any formal education, 175 (52.8%) had up to primary education and 19 (5.7%) schooled to the tertiary level. Economically, the findings indicated that 277 (83.7%) were employed and 54 (16.3%) were not employed. Majority 185 (55.9%) of participants earned less than 200 cedis ($25) per month with a few numbers eight (2.4%) of the participants earning more than 1500 cedis ($193) per month. Most 236 (71.3%) respondents took the bus to the facility with about 22 (6.6%) walking to the facility for treatment. One hundred and fourteen (34.4%) of participants spent less than 10 cedis ($1.29) as cost for travelling to the centre while about 79 (23.9%) spent over 50 cedis ($6.45) to travel to the centre as shown in Table 1. About 62% of the participants exhibited good level of medication adherence while about 37% showed poor adherence to medication (Table 1).

**Table 1:** Socio-demographic characteristics in relation to viral load non- suppression

**Demographic information**	**Frequency (%)**	**Viral load non- suppression**	
		**X**^**2**^	***P* value**
**Sex**		6.941	.331
Male	157 (47)		
Female	174 (53)		
**Marital status**		8.998	.169
Single	33 (10)		
Married	212 (64)		
Divorced	9 (3)		
Separated	42 (13)		
Widowed	7 (2)		
Cohabiting	28 (8)		
**Religion**		6.380	.223
Christian	223 (67.4)		
Muslim	103 (31.1)		
Traditionalist	5 (1.5)		
**Educational status**		4.271	.184
None	49 (15)		
Primary	60 (18)		
JHS	175 (52.8)		
SHS	28 (8.5)		
Tertiary	19 (5.7)		
**Employment status**		0.615	.022
Employed	277 (83.7)		
Unemployed	54 (16.3)		
**Age (years)**		20.750	.013
18–25	13 (3.9)		
26–35	52 (15.7)		
36–45	78 (23.6)		
46–55	95 (28.7)		
Above 55	93 (28)		
**Monthly Income**		10.637	.011
Less than 200GHS ($25)	185 (55.9)		
GHS200 – 500 ($25–64.5)	77 (23.3)		
GHS600 – 1000 ($77.4–129)	46 (13.9)		
GHS1001 – 1500 ($129.13–193.5)	15 (4.5)		
Above GHS1500 ($193.5)	8 (2.4)		
**Means of transport**		0.918	.040
By bus	236 (71.3)		
By foot	22 (6.6)		
Other	73 (22.1)		
**Cost of travelling to ART centre**		1.924	.017
Less than GHS10 ($1.29)	114 (34.4)		
GHS10 – 30 ($1.29–3.87)	97 (29.3)		
GHS31 – 50 ($6.45)	41 (12.4)		
More than GHS50	79 (23.9		
**Adherence to Medication**		3.776	< .001
Good adherence (compliant)	206 (62.2)		
Poor adherence (non-compliant)	125 (37.7)		

### Association between socio-economic factors and viral load non-suppression

Results from Pearson’s chi-square and Fisher’s exact test showed that there was no statistically significant difference between viral load non-suppression and sex (*p* = 0.331), marital status (*p* = 0.169), religious affiliation (*p* = 0.223), educational level (*p* = 0.184). However, there was a direct statistically significant difference between age (*p* = 0.013), employment status (*p* = 0.022), income (*p* = 0.011) and means of transportation (how patients travel from their community to the ART centre) (*p* = 0.040), cost of travel to the ART centre (*p* = 0.017) and level of medication adherence (*p* < 0.001) and viral load non-suppression as shown in Table 1.

### Proportion of PLHIV/AIDS on ART who are not achieving viral load suppression

**Fig. 1:**
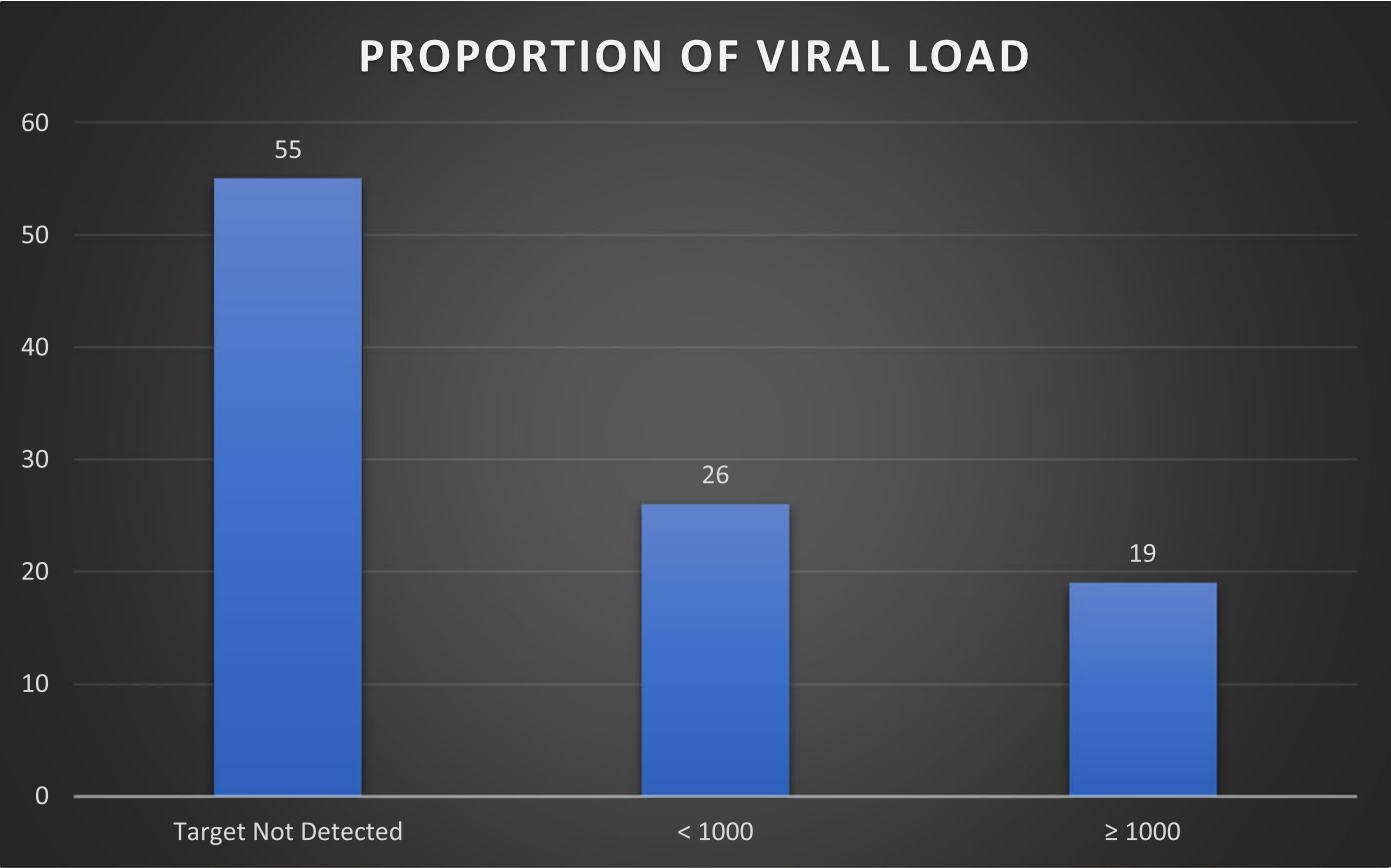
Proportion of PLHIV on ART who are not achieving viral load suppression

The findings from the study as indicated below in Fig. 1 show that 45% of PLHIV on ART had detectable viral loads while 55% PLHIV on ART viral loads were undetectable. In addition, 26% of PLHIV on ART exhibited a suppressed viral load while 19% of PLHIV showed unsuppressed viremia.

## Discussion

The study assessed the number of PLHIV/AIDS who are on ART and are viral load non-suppressed, as well as the determinants of viral load non-suppression among PLHIV/AIDS on active anti-retroviral therapy in Adansi South District, Ghana. We found that 19% of patients at the outpatient unit of the AIDS centre had viral load suppression. Pearson’s chi-square and Fisher’s exact test showed that employment, age, income, means of transportation and cost of travel to centre for ART was significantly associated with viral load suppression.

### Demographic characteristic of participants

The study revealed that most of the PLHIV were females as compared to the male population. The finding is in agreement with global data that women contribute the highest to the total population of PLHIV currently [21]. Most importantly women in low-middle-income countries contribute significantly to the HIV pandemic, especially those in southern and eastern Africa and some indigenous women in some communities in the world [22]. There are a lot of legal and social factors that negatively affect women, and their ability to make an informed choice and decisions that affect their health and total well-being. These expose vulnerable women to the risk of infection and limit access to effective, quality and affordable care as indicated by WHO, 2019 report.

### Prevalence of patients with viral load non-suppression

Virological failure among PLHIV is associated with the presence of comorbidities like active tuberculosis, mental conditions, high level of pre-treatment HIV RNA, and late initiation of ART [7, 23, 24]. A significant proportion of the study participants (19%) had virological non-suppression after being on ART for 12 months which is similar to the estimate made by Ghana with respect to its achievement of the third 90 of the UNAIDS 90–90–90 targets. Viral load non-suppression in our study was lower than (23.6%) reported by Ansah et al. (2021). The high rate of non-suppression suggests the need for intensified efforts to improve HIV treatment in PLHIV/AIDS to achieve the current 95–95–95 targets proposed by UNAIDS to end AIDS by 2030 [25].

Barriers to achieving the UNAIDS 2020 strategic target of 90% undetected viremia continues to pose a great deal of challenge to the success of the set goal, especially in the low-and-middle-income countries such as in Sub-Saharan Africa. Individual factors such as age, religion, educational status, adherence, and socioeconomic factors like poverty, not being married, stigmatization and disclosure continue to be a barrier to undetected viremia as indicated in the report by WHO (2016). A strong relationship between linkage and retention to care by PLHIV remains the hope of achieving undetected viremia in the HIV/AIDS continuum of care [26]. This current study revealed that the proportion of participants achieving viral non-suppression after 12 months on active antiretroviral therapy was 19%. The main reason for client’s enrolment on antiretroviral therapy is to suppress the replication of RNA of HIV to the barest minimum, halt the development of drug resistance viruses, restore and improve patients’ immunity, combat HIV/AIDS-related events and enhance the life span of the HIV-infected individual as reported by Bayu (2017), Bulage et al. (2017) and Musheke et al. (2013). Even though 19% is not huge, there is more room for improvement to ensure a reduction in the number of viral non-suppressed PLHIV. This can be achieved by identifying the factors or determinants responsible for the non-suppression.

The study revealed that age as a demographic factor of the PLHIV on the ART was the main factor that influence’s viral non-suppression. Various available studies support the fact that age has a significant impact on viral non-suppression [7, 27]. Young adults who are HIV-infected are less likely to be linked and retained in care as compared to adults, leading to high morbidity and mortality rates among young adults as a result of viral non-suppression [28]. The study revealed that 20.2% of the 18 -35 age bracket recorded viral non-suppression load. This is in agreement with available studies conducted in sub-Saharan Africa and Asia with a total population of 3934 supporting the fact that young adults are at risk of dropping out from care and recording a high level of viremia as compared to adults > 25 years old [29]. The challenges that are associated with HIV/AIDS such as anticipated stigma; rejection and sense of shame, fear of disclosure and loss of job, as well as stress have a greater impact on young adults as compared with the adults who have developed the capacity as a result of experience and resources to manage HIV-related stigma and stress [30, 31]. There are diverse opinions relating to young adult and adult viral suppression [26]. HIV-infected young adults’ experience is a worse form of immunological response and viral suppression as compared to adults leading to a high level of HIV-related morbidity and mortality in young adults especially in low-and-middle-income countries [32]. Intensifying the campaign on stigmatization can go a long way to reduce this canker.

### Socio-economic factors associated with viral load non- suppression

The study found that the major determinants of viral load non-suppression were socio-economic issues comprising; income (*p* = 0.02), employment (0.04), means of transportation (*p* = 0.02), and cost of transportation to the ART centre (*p* = 0.03). These findings support the assertion that the individual's economic status has a more significant impact on HIV infection and the success of adherence and attainment of viral suppression [33]. Economic constraints impede their ability to afford some hospital costs and payment of treatment in opportunistic infectious cases and other costs associated with care. It also affects their nutrition, accommodation, and safety of these HIV-infected individuals [34]. HIV infection is commonly associated with economically disadvantaged people [34, 35].

Most services already offer ARVs for free, removing the need to pay for them as a source of worry and anxiety for patients. The provision of free antiretroviral treatment, on the other hand, has not removed financial obstacles to adherence as found in this study. ART drugs and HIV services should be decentralized to the Community-based Health Planning Services centres and Health centres within their localities to decrease the burden of transportation. It will also curb loss of patients to follow-up due to giving of wrong addresses by patients and regular change of phone numbers since the Community Health Nurses are within their zone and will sometimes do home visits. However, factors such as the fear of stigmatization and mistrust of health staff within their locality may affect patients’ preference to travel kilometres for their drugs in spite of the tendency to default. Furthermore, most HIV/AIDS patients do not disclose their condition to their relatives [36, 37]; hence their inability to get support from relatives.

Transport costs for monthly clinical visits was identified as a potential barrier to ARV adherence in sub-Saharan Africa [38] with same confirmed by this current study. The shortage of transportation funding has been said to lead to missed doses and medical appointments. Conflicting requests for travel expenses and other essential items like food, accommodation and school fees could pose great economic burden on patients. Transportation costs could also jeopardize access to care. Interventions to overcome this barrier are crucial in Ghana and the rest of Sub-Saharan Africa for ART programmes to be successful.

Transport costs, especially those in rural areas, may be substantial compared to income and may compete with other major expenditures such as food, accommodation, and schooling. In several reports, transport costs were reported as a potential barrier to long-term antiretroviral treatment [8, 39–41]. Efforts to reduce the cost of transportation will help improve viral load non-suppression.

There was a significant association between adherence to medication and viral load non-suppression among PLHIV. This is in agreement with a study by Afrane et al. (2021) which identified level of medication adherence as a major factor contributing to viral non-suppression. The study by Afrane et al. (2021) was a cross-sectional study which included 250 children from the age of eight months to 15 years old. Despite the association of viral load non-suppression and level of medical adherence, it was recommended by the Ghana Health Service through the National AIDS control programme (2016), that individuals presenting with viral loads > 1000 copies/ml should undergo a strict ART adherence with monthly viral load test for three months to determine whether high viral load titres is due to poor adherence or treatment failure. Thus, viral load non-suppression in persons with good medication adherence could help identify treatment failure [42]. A comparative analysis of adherence in parts of Africa and Asia showed that adherence rates of medication was 92.7% in Africa and 95.2% in Asia [43]. This shows that the overall adherence rate of participants to medication was low in this study compared to previous studies. Another study by Opoku et al. (2022) revealed that 59.5% of the participants exhibited good medication adherence which is in line with the findings of this study. However, the levels of medication adherence were categorized into good, fair and poor, unlike this study which considered only good and poor adherence. The study by Opoku et al. was a retrospective study involving 720 participants in Komfo Anokye Teaching Hospital, Kumasi, Ghana.

### Limitation

The study was conducted in the Adansi South District in the Ashanti Region of Ghana with a population of less than 10% of the whole country’s population, therefore, generalizability should be done with caution. The cross-sectional study design used in this study is not as robust as a study design such as a cohort study or case–control study where two groups of participants could have been followed over a period prospectively for factors contributing to viral load non-suppression. Since some parts of the data were self-reported by the participants, there is the possibility of recall bias affecting the accuracy of the data collected. That notwithstanding, our study has revealed that factors such as income, cost of transportation and level of medication adherence influence viral non-suppression. This could be the basis for ART drugs and services’ decentralization to community health workers’ level within the various localities of patients to decrease the economic consequences involved in accessing health care for PLHIV/AIDS. This will minimize defaulting, improve adherence and promote viral load suppression in PLHIV/AIDS.

## Recommendations

It is recommended that future studies should focus on the use of a larger sample drawn from other regions and districts of the country to ensure generalizability of study findings. Future studies should consider a more robust study design such as cohort or case–control to improve the efficiency and accuracy of the study since possible bias and confounders can be well controlled. ART drugs and services should be decentralized to the community health workers’ level within the various localities of patients to decrease the economic consequences involved in accessing health care for PLHIV/AIDS. This will minimize defaulting, improve adherence and promote viral load suppression.

## Conclusion

There was a low level of viral non-suppression among PLHIV after 12 months of active antiretroviral therapy with age, income, employment, means of transportation, cost of transportation and level of medication adherence influencing viral non-suppression. A review of ART services to ensure availability at the community level will also promote viral load suppression.

## Data Availability

The datasets used and/or analysed during the current study are available from the corresponding author on reasonable request.

## Abbreviations

HIV: Human immunodeficiency virus
ART: Anti-retroviral therapy
PLHIV: People living with HIV
AIDS: Acquired immunodeficiency virus syndrome
SSA: Sub-Saharan Africa
